# Supplementary material for: Accelerating Density Functional Calculation of Adatom Adsorption on Graphene via Machine Learning
Source: Materials (Basel). 2023 Mar 26;16(7):2633. doi: 10.3390/ma16072633 (PMC10095669; doi:10.3390/ma16072633)
Supplement: Supplementary file 1 [file materials-16-02633-s001.zip › Supplementary Material_S1.pdf]

Supplementary Material

Accelerating Density Functional Calculation of Adatom Adsorption on Graphene via Machine Learning

Nan Qu [1,†], Mo Chen [1,†], Mingqing Liao [1], Yuan Cheng [2], Zhonghong Lai [3], Fei Zhou [1], Jingchuan Zhu [1,*], Yong Liu [1,*] and Lin Zhang [4]

[1] School of Materials Science and Engineering, Harbin Institute of Technology, Harbin 150001, China
[2] National Key Laboratory of Science and Technology on Advanced Composites in Special Environments, Harbin Institute of Technology, Harbin 150001, China
[3] Center of Analysis, Measurement and Computing, Harbin Institute of Technology, Harbin 150001, China
[4] Biological Physics, Department of Physics and Astronomy, University of Manchester, Oxford Road, Manchester M13 9PL, UK
* Correspondence: fgms@hit.edu.cn (J.Z.); lyonghit@hit.edu.cn (Y.L.)
† These authors contributed equally to this work.

Supplementary Table S3. Parameters used in genetic algorithm.

genetic Algebra	30
population size	50
crossover probability	0.5
mutation probability	0.2

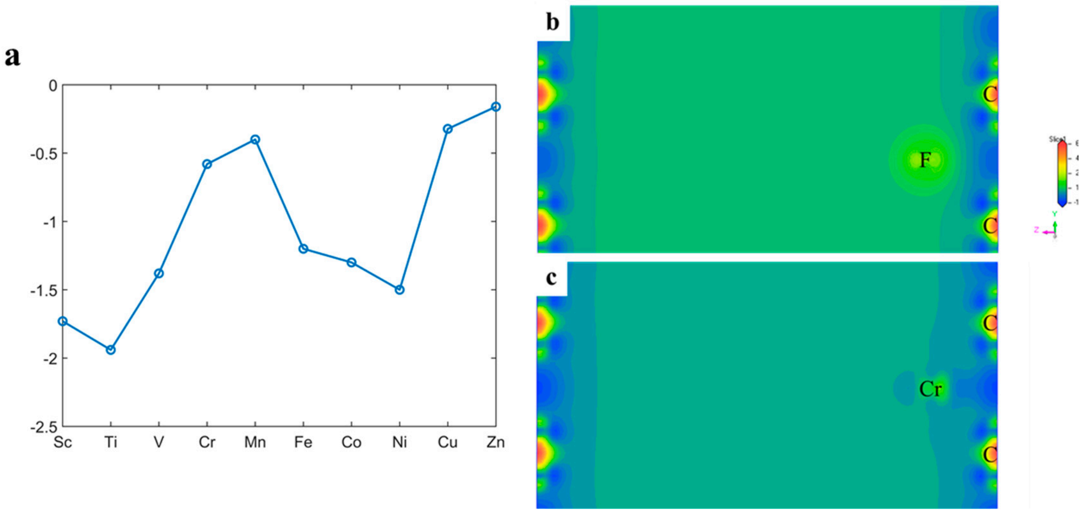

Supplementary Figure S1. (a) Evolution along the 3d series according to DFT prediction, and the electron density difference plots of (b) F and (c) Cr adatom adsorption on graphene.

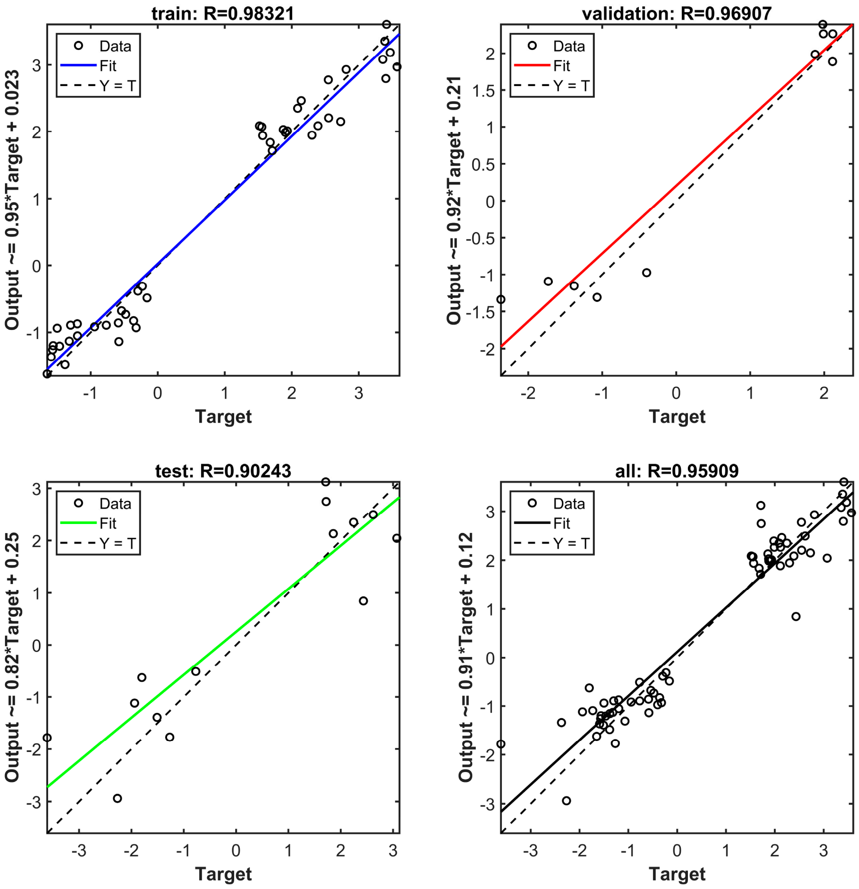

Supplementary Figure S2. Prediction performance plots with train, validation, test and the all dataset.

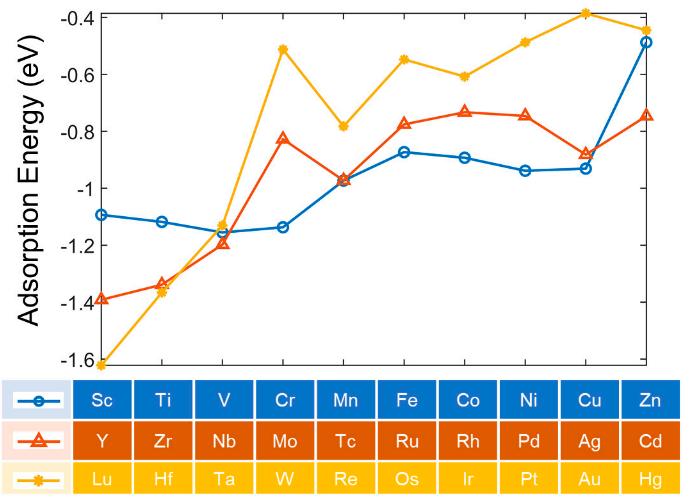

Supplementary Figure S3. Evolution transition metal according to ML prediction.

Supplementary Box S1.

The GGA in PBE format and the PAW method are employed. A plane wave basis with a cut-off energy of 380 eV and "3×3×1" k-sampling in Brillouin zone are used. The Graphene/Mg interfaces are shown in supplementary Figure S4. Mg atoms cut off along Mg (0 0 0 1) facet are used as the metal matrix with one graphene layer. The vacuum layers of 10 Å is used. Cohesive energy (E_{coh}) is calculated as

$$E_{coh} = \frac{1}{a+b+c}\left(aE_{atom}^{C} + bE_{atom}^{Mg} + cE_{atom}^{M} - E_{\text{total}}^{Gra\,/Mg+M}\right)$$

where a, b and c are the number of atoms, E_{atom} is the energy of single atom, and $E_{\text{total}}^{Gra\,/Mg+M}$ is the energy of graphene/Mg+M interface.

Supplementary Table S5. Bond population analysis of Graphene/Mg interface. The bonds with the strongest bond strength are as follows.

Interface	Bond	Population	Length (Å)
Graphene/Mg+Al	C-Al	--	--
	C-Mg	--	--
	Mg6-Al2	0.11	2.95
Graphene/Mg+Zn	C-Zn	--	--
	C-Mg	--	--
	Mg15-Zn3	-0.20	2.85
Graphene/Mg+Ca	C15-Ca1	-0.07	2.80
	C-Mg	--	--
	Mg-Ca	--	--
Graphene/Mg+Li	C-Li	--	--
	C-Mg	--	--
	Mg5-Li1	-0.22	2.91

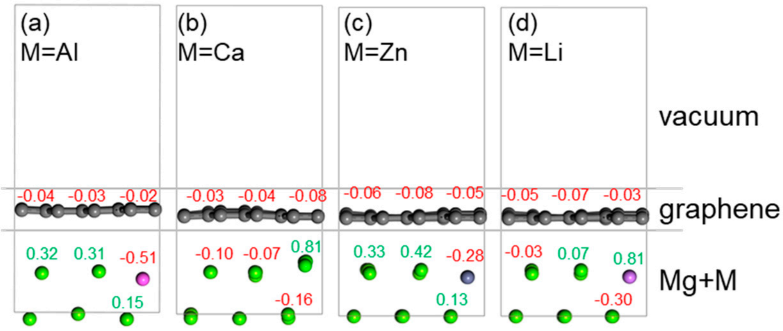

Supplementary Figure S4. Mulliken Population analysis of Graphene/Mg+M interfaces.

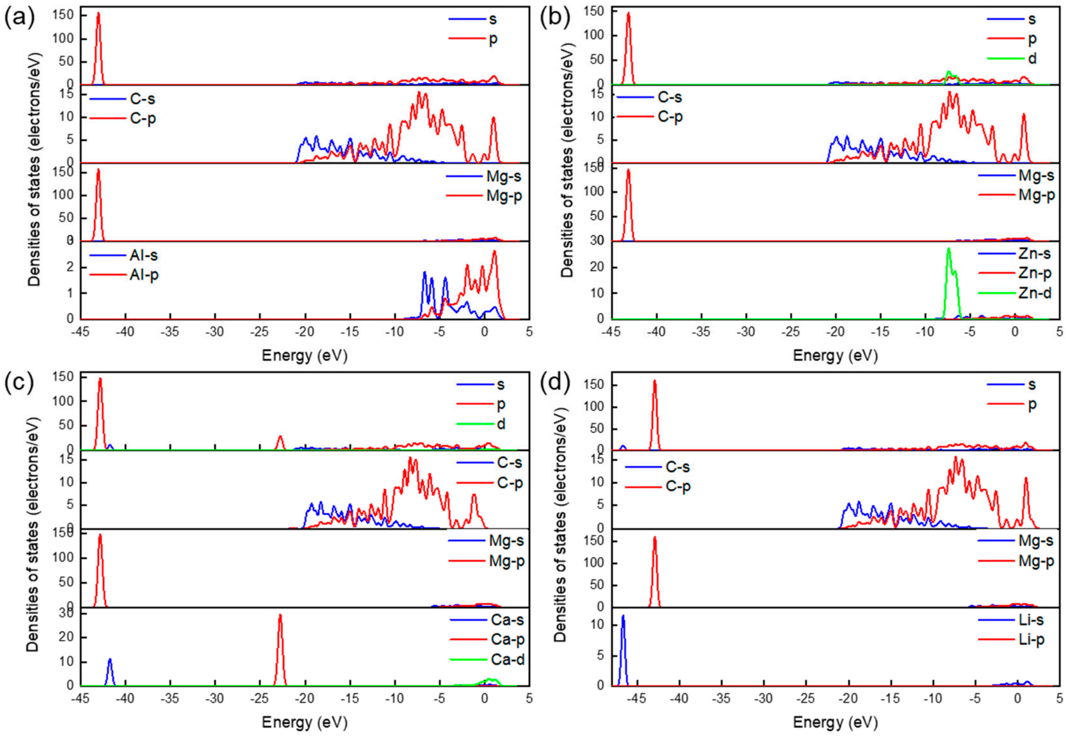

Supplementary Figure S5. Partial density of states (PDOS) for Graphene/Mg interface (a) Mg+Al, (b) Mg+Zn, (c) Mg+Ca and (d) Mg+Li.

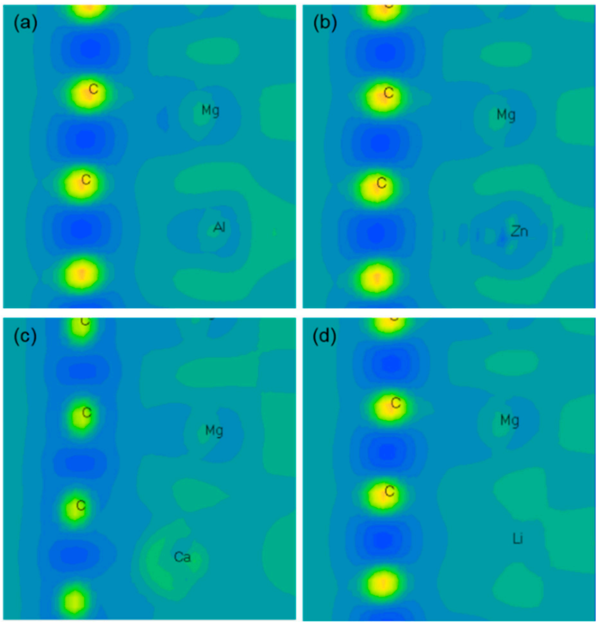

Supplementary Figure S6. Electron density difference of Graphene/Mg interface (a) Mg+Al, (b) Mg+Zn, (c) Mg+Ca and (d) Mg+Li.
